# Diagnostic Obscurity of Gastrointestinal Subepithelial Tumors: An Organizing Gastric Hematoma Requiring *En Bloc* Resection

**DOI:** 10.14309/crj.0000000000001024

**Published:** 2023-04-10

**Authors:** Clive Jude Miranda, William Schertzing, Anushi Shah, Paul Anthony Reyes Del Prado

**Affiliations:** 1 Department of Surgery, Valleywise Health Medical Center, Phoenix, AZ

**Keywords:** subepithelial tumor, chronic hematoma, exophytic gastric mass

## Abstract

Gastric subepithelial tumors (SETs) are often incidentally found during examinations of the gastrointestinal tract. Able to arise from any layer of the stomach, these tumors are predominantly asymptomatic and are classified as either benign or malignant based on size, consistency, shape, and mobility as determined by endoscopic evaluation. We present the first reported case of a gastric SET presenting as a chronic organizing hematoma. In doing so, we discuss the necessity of multimodal imaging techniques and multidisciplinary management in identifying often obscure gastric SETs to intervene on potentially malignant masses early.

## INTRODUCTION

Subepithelial tumors (SETs) are frequently encountered in the gastrointestinal tract, the majority of which are asymptomatic and discovered incidentally. Characterized by location, size, and echogenicity, the gold standard of diagnosis for SETs is histology and immunohistochemistry, usually performed with fine needle aspiration. There is a spectrum of imaging modalities to diagnose SETs with 2 major ones being esophagogastroduodenoscopy (EGD) and endoscopic ultrasound (EUS). EGD is suboptimal in differentiating intraluminal lesions from extraluminal SETs with a sensitivity and specificity of 87% and 29%, respectively. By contrast, EUS is the most accurate method for differentiating SET location. They are operator-dependent, but, in the right hands, EUS approaches a sensitivity of 92% and a specificity of 100%. Despite this, some cases require other modalities to make a definitive diagnosis.^1–3^ Symptomatic masses should be resected independent of diagnosis.^1,4,5^ In the case that a biopsy is inconclusive, imaging along with symptomatology is used to guide operative intervention.^1,6^ Here, we highlight the concurrent necessity yet limitation of multimodal imaging techniques for the diagnosis of gastric SETs as well as present a first-reported finding of an enlarging gastric mass as an organizing, chronic, intraluminal hematoma.

## CASE REPORT

**Figure 1.**
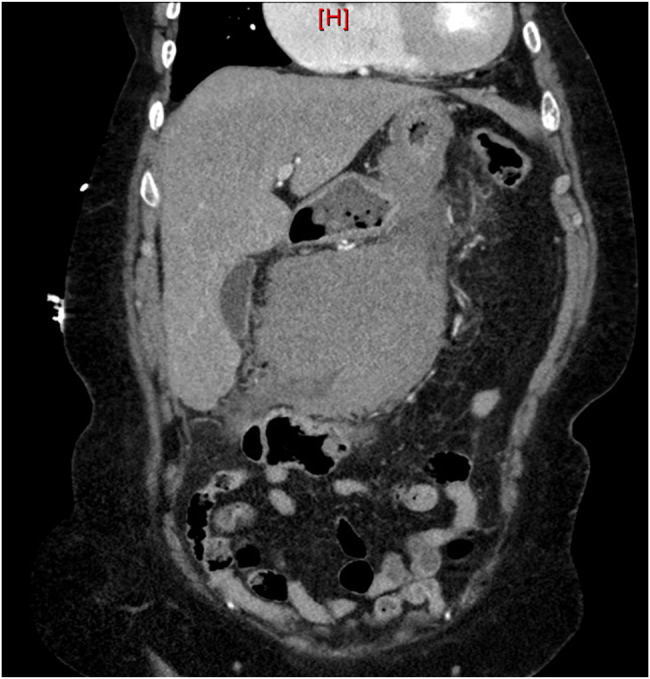
Computed tomography of the abdomen/pelvis with intravenous contrast: large exophytic mass arising from the greater curvature of the stomach. Gastrointestinal stromal tumor favored.

**Figure 2.**
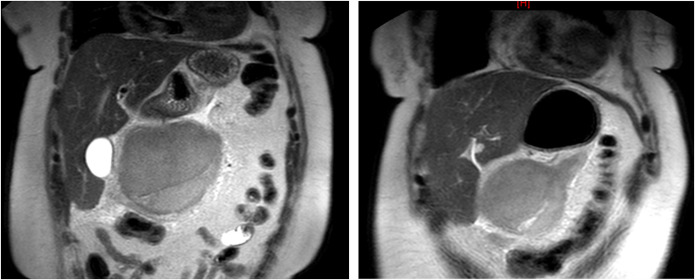
Magnetic resonance imaging of the abdomen with and without contrast: 13 × 10-cm circumscribed, hypovascular mass located inferior to the stomach with adjacent fat stranding, possibly arising from the greater curvature of the stomach. Distinct from the pancreas and colon. Likely differential and considerations include gastrointestinal stromal tumor, among other etiologies such as leiomyoma.

A 64-year-old woman presented with emesis and worsening epigastric pain for 24 hours. Medical history included uncontrolled type 2 diabetes, stage 4 chronic kidney disease, and a hiatal hernia. 30 years ago, she underwent an exploratory laparotomy with splenectomy for multiple abdominal gunshot wounds and a hysterectomy with bilateral salpingo-oophorectomy for fibroid tumors. Computed tomography (CT) of the abdomen/pelvis without contrast showed a large 12-cm mesenteric neoplasm arising from the transverse mesocolon without evidence of obstruction (Figure 1). We initially favored nonaggressive neoplasms and recommended bowel rest. Repeat CT with contrast showed an exophytic mass arising from the greater curvature of the stomach, favoring a gastrointestinal stromal tumor. Contrast allowed for improved structure identification and aided in differentiating endophytic vs exophytic characteristics. Magnetic resonance imaging of the abdomen showed a 13 × 10-cm circumscribed, hypovascular gastric mass with adjacent fat-stranding (Figures 2).

**Figure 3.**
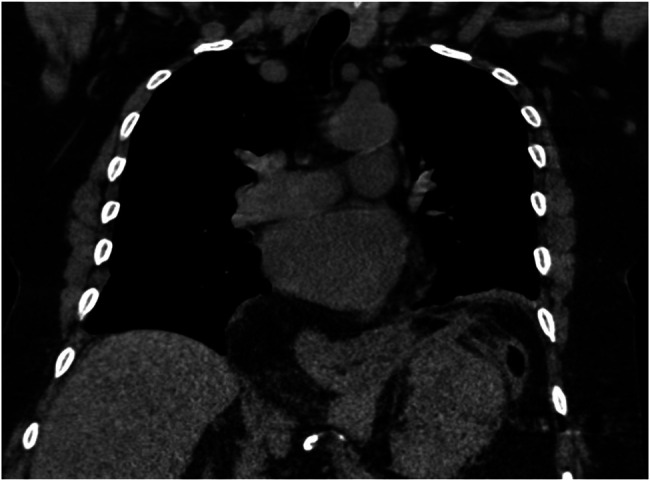
Computed tomography of the chest without contrast: performed 4 years earlier for another separate indication with incidental finding of abdominal mass captured in upper abdominal cuts of imaging.

Notably, a CT of the chest from 4 years earlier captured the upper abdomen, which did show the mass being present, but no workup was performed for it (Figure 3). EGD showed a 4-cm hiatal hernia, erosions in the antrum and mass effect on the second portion of the duodenum. EUS showed an 8 × 5-cm hypoechoic mass extending from the fourth layer of the greater curvature of the stomach. Fine needle aspiration was performed with no obvious core tissue seen and a maroon clot-like material removed. Because of the nonobstructing nature of the mass, the patient felt better after 48 hours of bowel rest and tolerated a diet before discharge. Pathology showed adipose tissue with rare, atypical cells and organizing blood fragments that were insufficient for definitive diagnosis.

**Figure 4.**
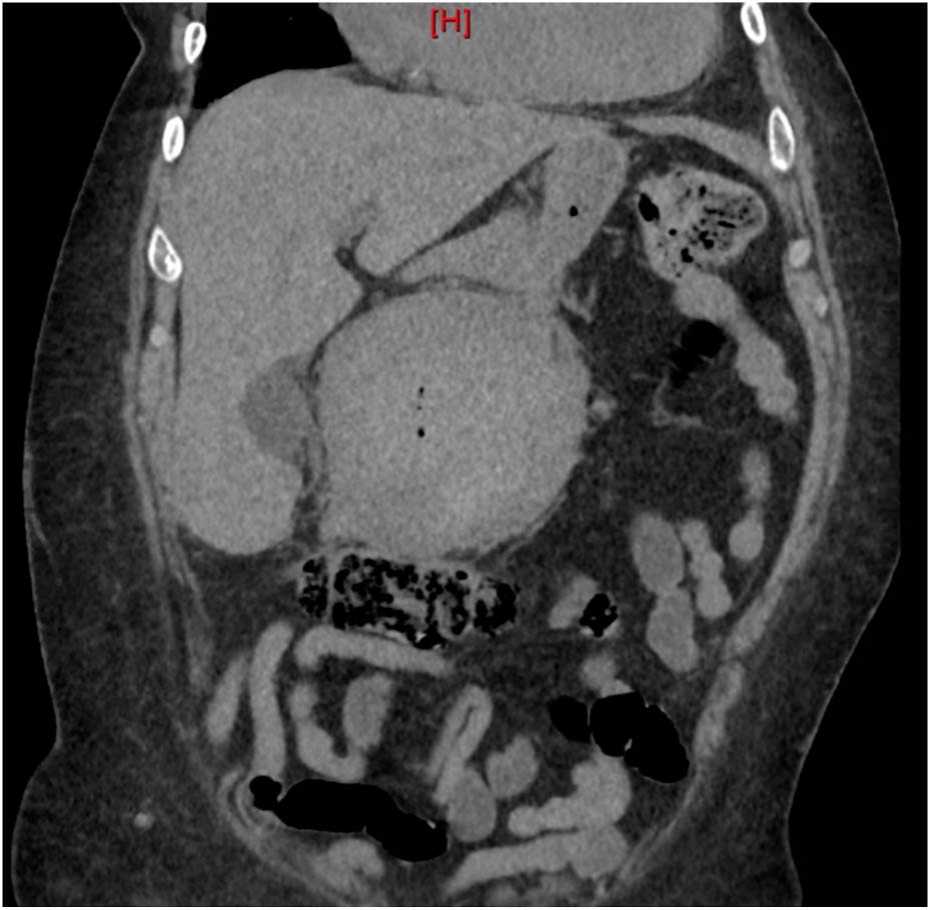
Computed tomography of the abdomen/pelvis without contrast: mild interval increased size of a hyperdense perigastric mass, recently biopsied. Previously favored to represent an exophytic gastric neoplasm given presence on previous chest computed tomography examination from 4 years earlier. However, recurrent lesser sac hematoma is a likely etiology, given appearance on recent endoscopy and imaging.

**Figure 5.**
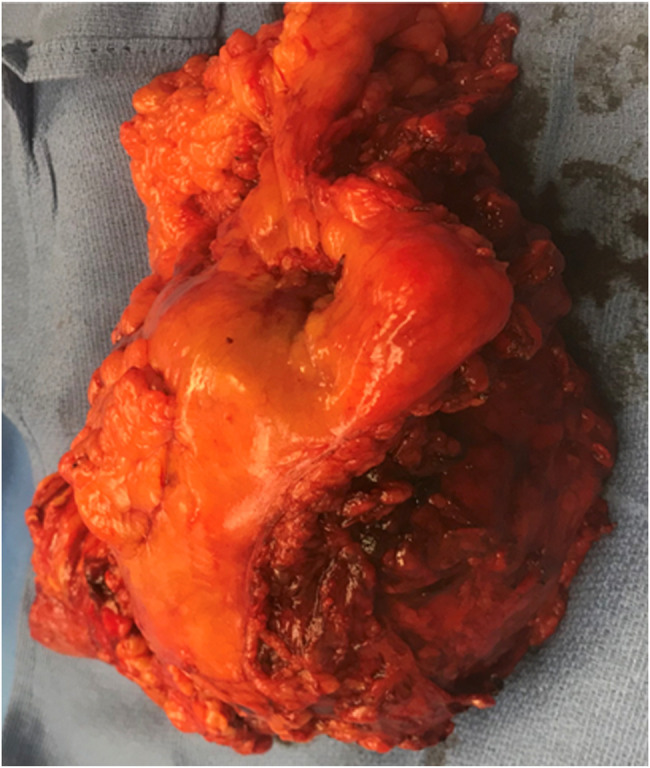
*En bloc* resection of mass adherent to stomach and transverse colon mesentery.

She presented to clinic several days later, however, with unrelenting epigastric pain and was immediately sent to the emergency department where a CT showed an increased size of a hyperdense perigastric mass (Figure 4). The patient underwent an exploratory laparotomy, and after extensive lysis of adhesions, an exophytic greater curvature mass with adhesions to the pancreas, duodenum, liver, small bowel, and large bowel was encountered with involvement of transverse colon mesentery. Because of no confirmed pathology, an oncologic resection with formal lymphadenectomy was not performed with the acknowledgement that a subsequent resection may be needed based on final pathology results. An *en bloc* resection was performed with an open extended right hemicolectomy and partial gastrectomy with stapled, side-to-side antiperistaltic ileocolonic anastomosis. Postoperative course was unremarkable, and the patient was discharged to a nursing facility on postoperative day 4. Pathology showed an organizing 11-cm hematoma with surrounding fibrosis (Figure 5). Multiple sections had fibroblastic proliferation and associated hemorrhage. Immunoperoxidase stains for CD117 and CD34 were negative in spindle cells. Iron, smooth muscle action, desmin, and calponin stains were positive. Stains for discovered on GIST-1 protein, anaplastic lymphoma kinase, and beta-catenin were negative. Sections of the gastric wall and colon revealed normal mucosa and bowel wall. The overall findings were consistent with an organizing chronic, intraluminal hematoma with fibrosis.

## DISCUSSION

To the best of our knowledge, this is the first case report regarding an enlarging gastric mass as an organizing intraluminal hematoma. Other cases in the literature have described acute gastric hematomas discovered endoscopically, intraoperatively, or on imaging. Ours was a case of spontaneous intraluminal hematoma associated with previous abdominal trauma and surgeries. Our patient presentation was different to other cases in the literature, and the identification of a hematoma was not as clear. Our working diagnosis was a tumor as the mass was seen 4 years earlier on another CT. Our imaging in this patient was indeterminate. The mass seemed to have some features of a hematoma but was concurrently noted to have no hyperintense T2 signal and absent calcification, both of which are classic imaging findings of chronic hematoma formation. Her repeat noncontrast CT, however, showed a hyperintense perigastric mass, an imaging finding that can be seen with hemorrhage but also with hypercellular neoplastic processes. The location at the greater curvature of the stomach and its continual increase in size, however, favored a neoplastic formation. It is also important to note that postcontrast subtraction imaging on magnetic resonance imaging was not performed. If this had been performed, one could have confirmed if the mass is intrinsically hyperintense from hemorrhage or protein content or a hypovascular mass with little enhancement. Pathology, however, was more definitive in the diagnosis of hematoma formation, considering the patient's medical history and recent trauma. The negative staining for CD117, CD34, and discovered on GIST-1 protein rules out gastrointestinal stromal tumor, Negative beta-catenin staining eliminates fibromatosis and negative ALK staining eliminates inflammatory myofibroblastic tumor as possible diagnoses. The positive smooth muscle action and desmin stains favor myofibroblastic cells and suggest a non-neoplastic process, and the overall pathology findings are consistent with fibrosis surrounding an organizing hematoma. The mass was indeed examined intraoperatively too. Further discussion as to the cause and possibility of malignancy associated with the chronicity of the mass was held until final pathology returned. gastrointestinal stromal tumor was not confirmed on staining, so succinate dehydrogenase testing was not performed. It is our theory that the patient's remote history of an exploratory laparotomy and splenectomy may have played a role in the initial development of a hematoma that never fully resorbed. She was not on any anticoagulation. Therefore, a slow growth, rather than an acute herald bleed, could be explained by our patient's other medical comorbidities precluding adequate healing of the hematoma. It is important to use multiple diagnostic tools to help guide operative intervention for subepithelial gastric masses with surgery being the best definitive treatment option in symptomatic patients.^5^

## DISCLOSURES

Author contributions: CJ Miranda, W. Schertzing, A. Shah, and PAR Del Prado compiled a literature review and wrote the manuscript. CJ Miranda is the article guarantor.

Financial disclosure: None to report.

Previous presentation: This case was presented at the American College of Gastroenterology; October 23, 2022; Charlotte, NC; and received an Outstanding Poster Presenter Award.

Informed consent was obtained for this case report.
